# Supplementary material for: Effects of lipid membranes on RNA catalytic activity and stability
Source: Biol Cell. 2025 Feb 26;117(2):e202400115. doi: 10.1111/boc.202400115 (PMC11865690; doi:10.1111/boc.202400115)
Supplement: Supplementary file 1 — Supporting Information [file BOC-117-e202400115-s001.pdf]

Effects of lipid membranes on RNA catalytic activity and stability - Supplementary data

Authors: Tomasz Czerniak[a], James P. Saenz[a, b*]

[a] Technische Universität Dresden, B CUBE Center for Molecular Bioengineering, 01307 Dresden, Germany

[b] Technische Universität Dresden, Faculty of Medicine, Dresden 01307, Germany

*corresponding author and lead contact: james.saenz@tu-dresden.de

Supplementary Table 1: T7 transcription templates (5' -> 3') and buffer components (**bold**: T7 promoter)

Schistosoma hammerhead DNA template for T7 transcription	GGAGGGCATTTCGTCCTATTTGGGACTCGTCAGCTGGATGTACCTCGC**CTATAGTGAGTCGTATTAG**
Sequence of HH-tRNA-HDV DNA template	**TAATACGACTCACTATAG**GGAGAAATCCGCCTGATGAGTCCGTGAGGACGAAACGGTACCCGGTACCGTCGCGGATTTAGCTCAGTTGGGAGAGCGCCAGACTGAAGATCTGGAGGTCCTGTGTTCGATCCACAGAATTCGCAGGGTCGGCATGGCATCTCCACCTCCTCGCGGTCCGACCTGGGCTACTTCGGTAGGCTAAGGGAGAAGCTTGGCACTGGCCGTCGTTTAAGGGCGAATTCTGCAGAT
Substrate of Schistosoma hammerhead	6-FAM-GGAGGGCAUCCUGGAUUCCACUCGCC
T7 transcription buffer	40 mM Tris pH 8<br>23.5 mM $MgCl_2$<br>15 mM DTT<br>10 mM NaCl<br>2 mM spermidine<br>7.5 mM nucleotides (1.88 mM each)<br>5 U/ml inorganic pyrophosphatase (NEB, M0361)<br>0.018 g/L T7 polymerase (homemade, MPI-CBG)

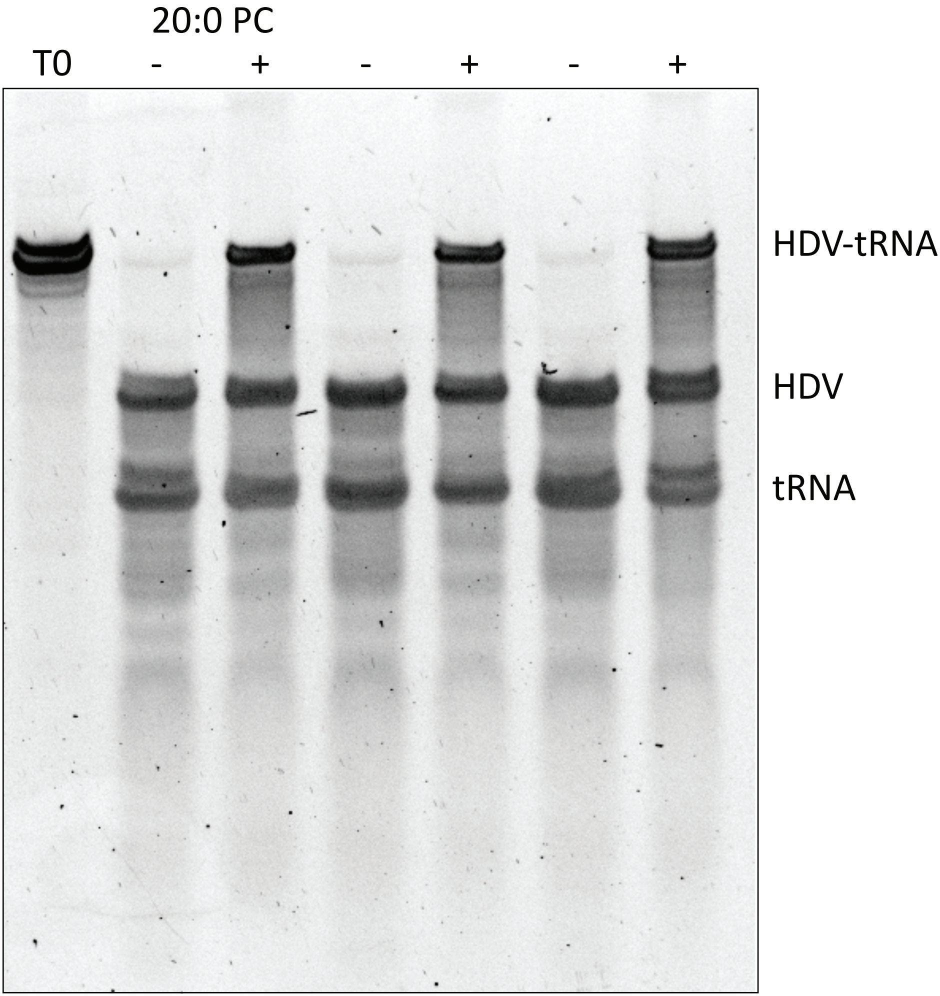

Supplementary Figure 1 HDV ribozyme is partially inhibited by lipid gel membranes after overnight temperature cycling (150 cycles). The reaction was performed in the buffer with 5 mM 20:0 PC (gel membranes), reaction triplicates are presented.

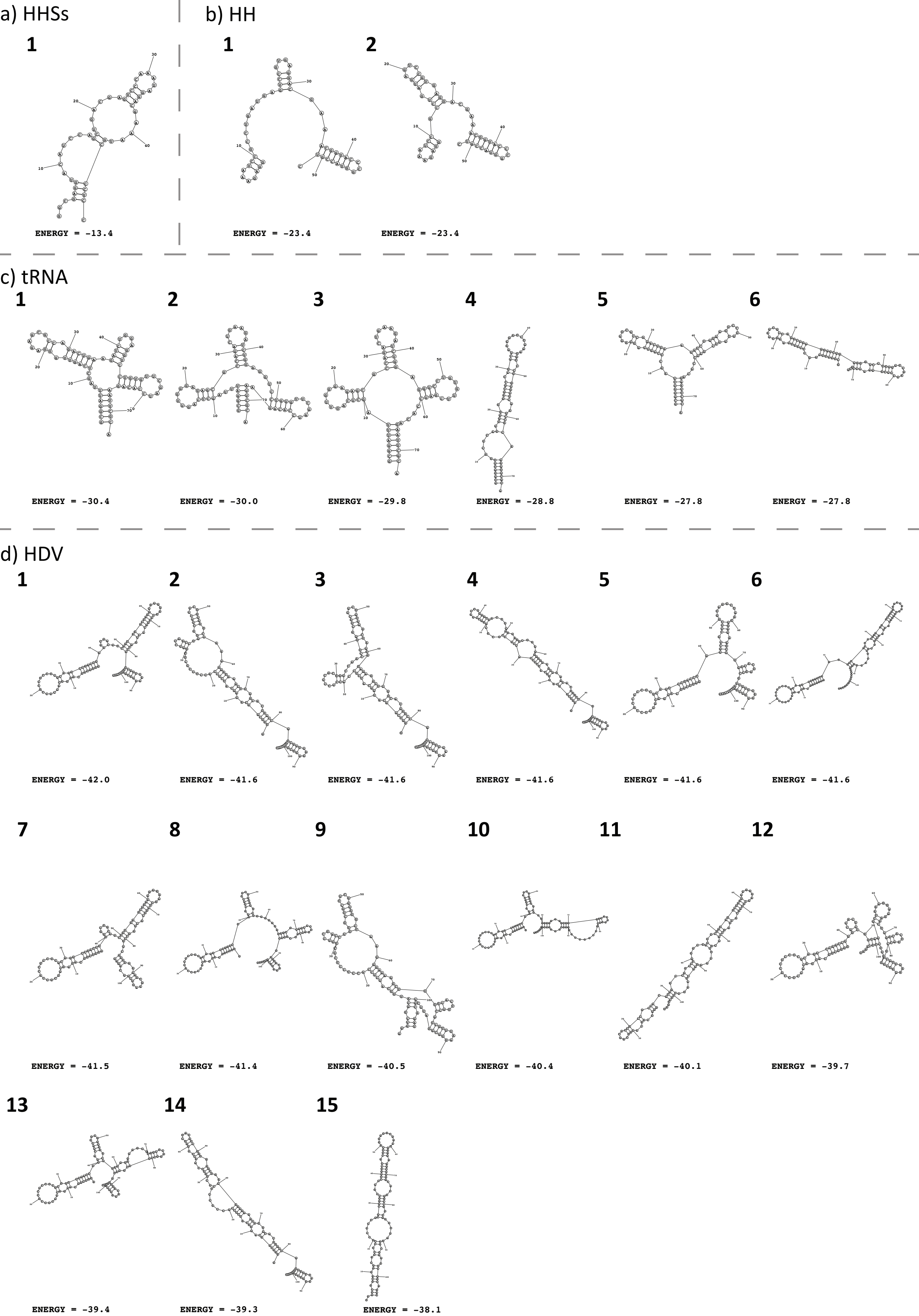

Supplementary Figure 2 Secondary structures of used RNAs generated in silico using fold tool[70].
a) Schistosoma hammerhead (HHSs) used as trans-acting ribozyme generates one dominant structure. **b)** Hammerhead generates similar two structures. **c)** tRNA generates multiple (6) different structures, dominantly double stranded. **d)** HDV generates the largest number of different structures (15), mostly double stranded. HH, tRNA, and HDV are part of the HH-tRNA-HDV construct. Presented energy values are free energy of structures in kcal/mol.

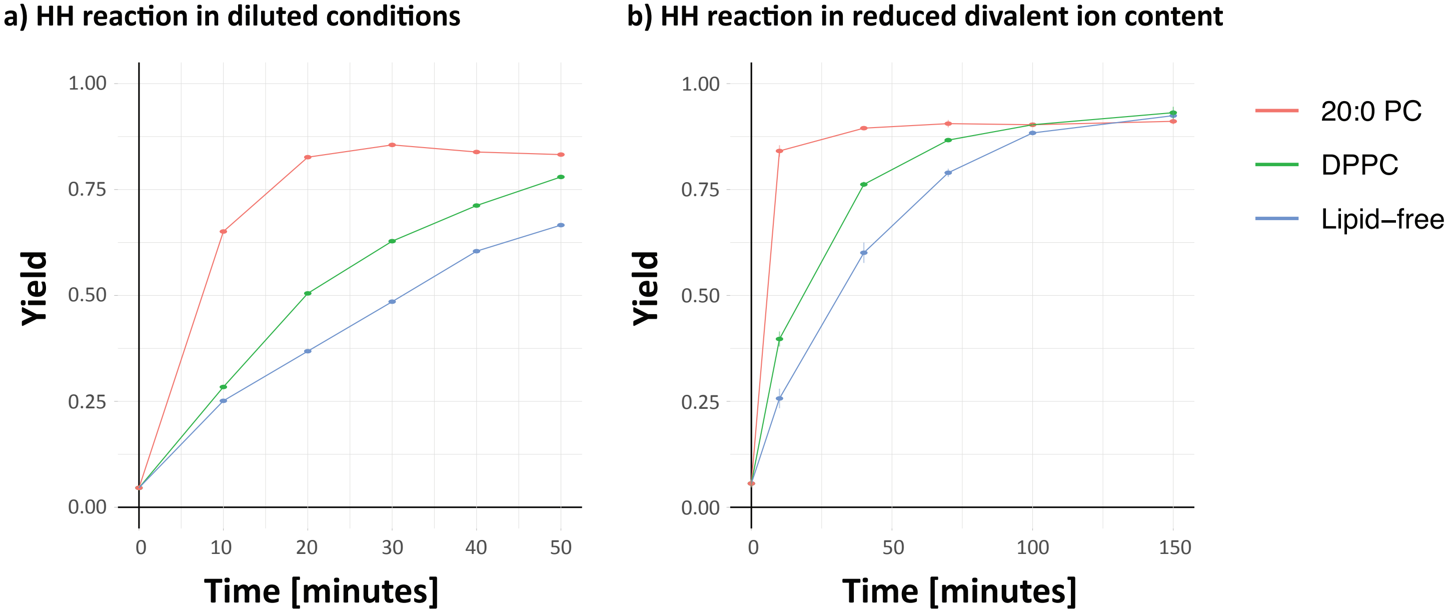

Supplementary Figure 3 HH ribozyme reaction rate increases in the presence of lipid membranes. a) Test experiment (n=1) of HH reaction in the diluted conditions (5 nM ribozyme, 10 nM substrate) shows that the presence of lipid gel membranes improves reaction rate; the largest effect was visible after 20-40 minutes. This experiment was performed once to determine the time point in which the differences of the HH reaction yield were visible for the lipid and lipid-free system, thus lacking error bars. **b)** HH reaction rate in the decreased divalent ion content (500 µM $MgCl_2$, 500 µM $CaCl_2$) is faster in the presence of lipid gel membranes and the largest differences are visible after 10-40 minutes of the temperature cycling (1-4 cycles). Error bars are SEM from 4 replicates and are relatively small (<3%), which might bias their readout.

a) Degradation of HDV ribozyme in the presence of lipid liquid membranes

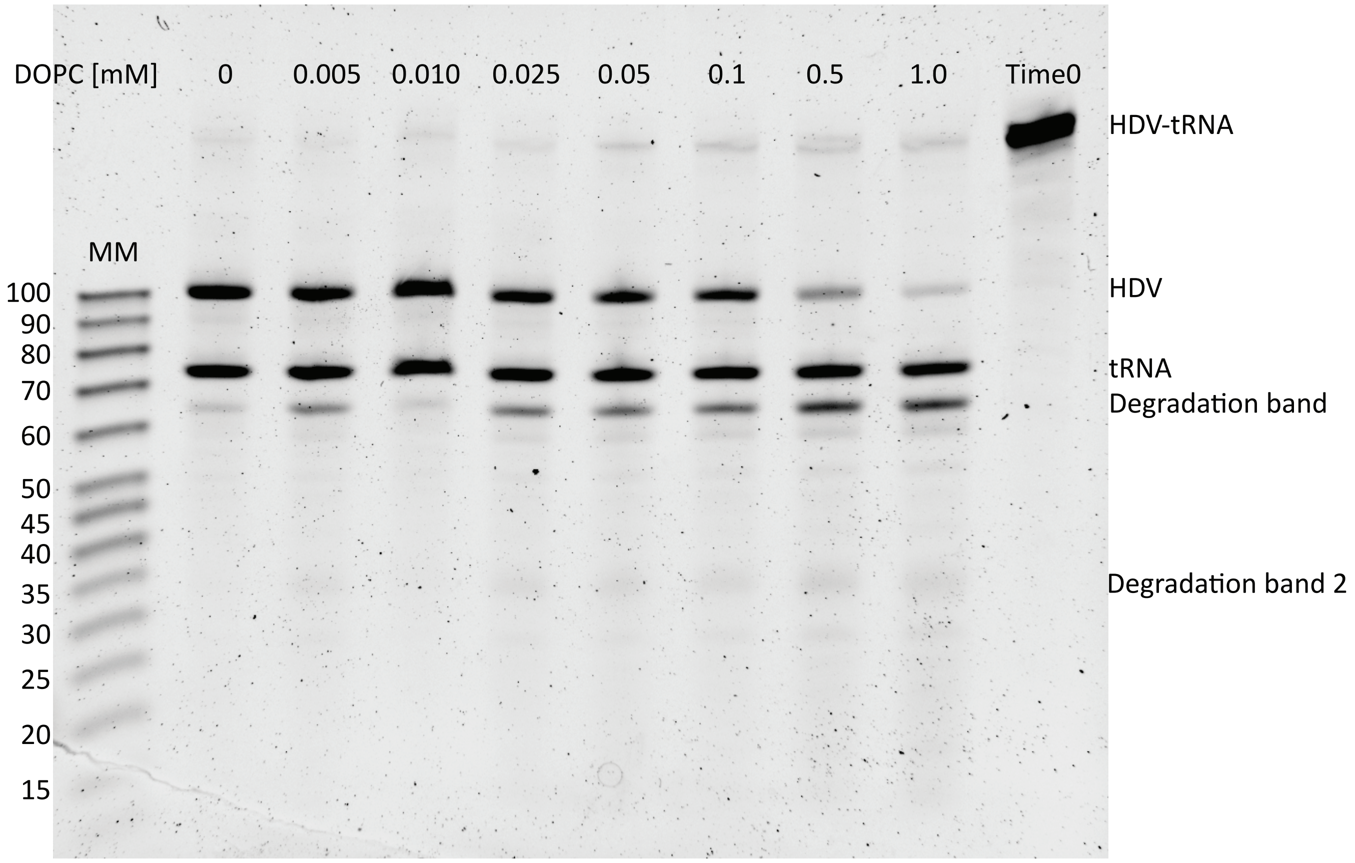

b) Length estimation of the degradation products

length [nt]	log length	migration [pixels]
100	2	509
90	1,95424251	616
80	1,90308999	738
70	1,84509804	884
60	1,77815125	1058
50	1,69897	1265
45	1,65321251	1381
40	1,60205999	1520
35	1,54406804	1671
30	1,47712125	1834
25	1,39794001	2016
20	1,30103	2234

RNA	migration [pixels]	Approx. Length
HDV	506	**99,9175894**
tRNA	833	**74,0541924**
Degradation1	982	**64,6058909**
HDV	512	**99,3699184**
tRNA	837	**73,7833393**
Degradation1	984	**64,4876347**
Degradation2	1715	**33,0109861**

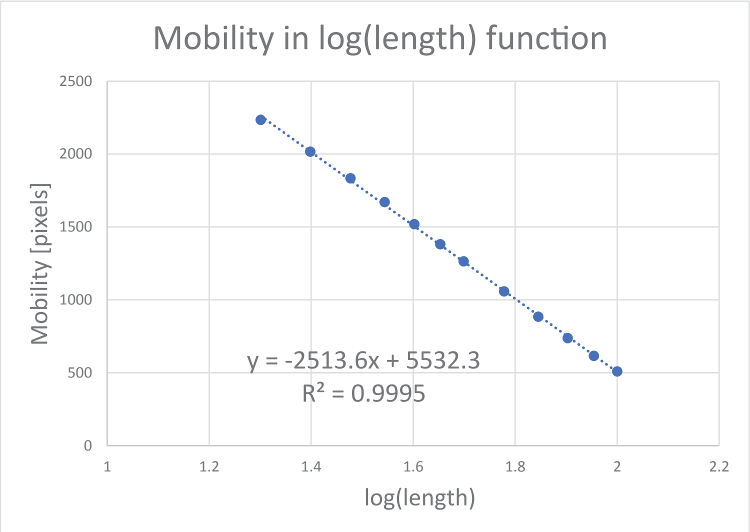

Supplementary Figure 4 HDV ribozyme degrades in the presence of lipid liquid membranes in a lipid concentration dependent manner. a) 20 ng of HDV-tRNA construct was incubated with various concentrations of DOPC lipid membranes (liquid lipid membranes) in temperature cycling conditions. RNA was then analysed using PAGE (run along with MM - length marker, Affimetrix, Ref: J76410, Lot: 4310399), presented lengths are in nucleotides. **b)** The marker migration was quantified as pixels in the whole lane, and the plot of migration (pixels) in the function of log(length) was generated ($R^2 = 0.9995$). Using the migration values for different bands and using a linear fit of the marker bands (graph) the lengths of the RNA bands presented on the gel were calculated. The degradation band 1 has a length ~ 65 nucleotides, whereas degradation band 2 ~ 35 nucleotides, which sums up to HDV length ~ 100 nucleotides.

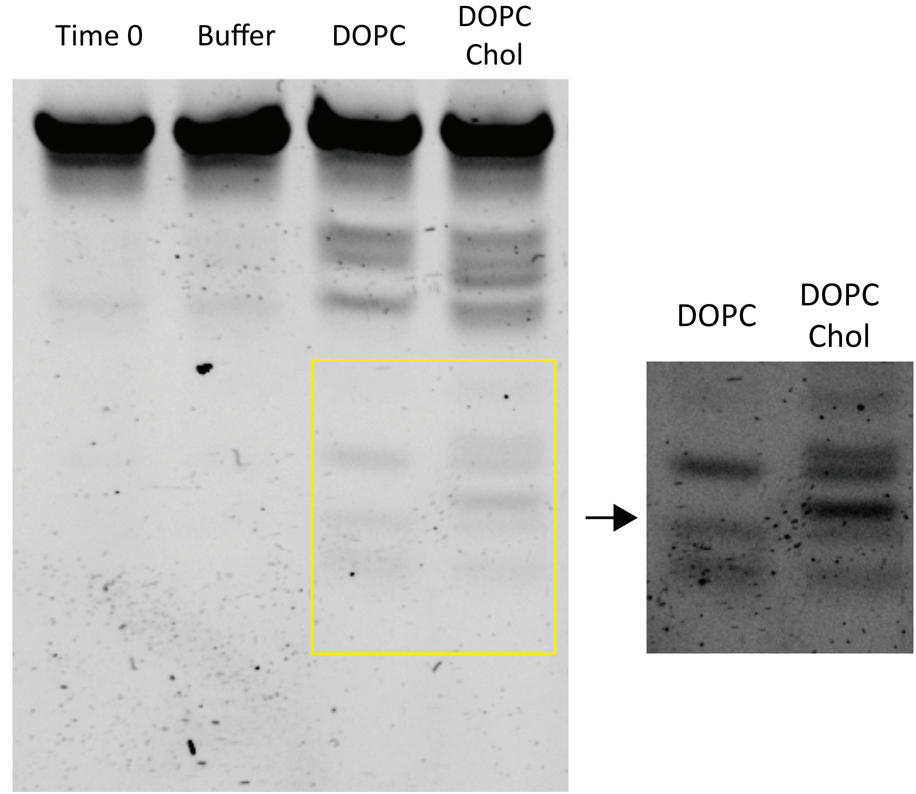

Supplementary Figure 5 The HH degradation pattern is different for different liquid membranes. HH was incubated with DOPC and DOPC:Chol membranes: both lipid membranes degrade HH, however the generated degradation pattern differs.
